# Bayesian penalized likelihood PET reconstruction impact on quantitative metrics in diffuse large B-cell lymphoma

**DOI:** 10.1097/MD.0000000000032665

**Published:** 2023-02-10

**Authors:** Jason R. Young, Vamshi K. Mugu, Geoffrey B. Johnson, Eric C. Ehman, Annie T. Packard, Andrew C. Homb, Mark A. Nathan, Gita Thanarajasingam, Bradley J. Kemp

**Affiliations:** a Department of Radiology, Mayo Clinic, Rochester MN; b Department of Immunology, Mayo Clinic, Rochester MN; c Department of Hematology & Oncology, Mayo Clinic, Rochester MN.

**Keywords:** BPL, Deauville, lymphoma, PET, Q.Clear

## Abstract

Evaluate the quantitative, subjective (Deauville score [DS]) and reader agreement differences between standard ordered subset expectation maximization (OSEM) and Bayesian penalized likelihood (BPL) positron emission tomography (PET) reconstruction methods. A retrospective review of 104 F-18 fluorodeoxyglucose PET/computed tomography (CT) exams among 52 patients with diffuse large B-cell lymphoma. An unblinded radiologist moderator reviewed both BPL and OSEM PET/CT exams. Four blinded radiologists then reviewed the annotated cases to provide a visual DS for each annotated lesion. Significant (*P* < .001) differences in BPL and OSEM PET methods were identified with greater standard uptake value (SUV) maximum and SUV mean for BPL. The DS was altered in 25% of cases when BPL and OSEM were reviewed by the same radiologist. Interobserver DS agreement was higher for OSEM (>1 cm lesion = 0.89 and ≤1 cm lesion = 0.84) compared to BPL (>1 cm lesion = 0.85 and ≤1 cm lesion = 0.81). Among the 4 readers, average intraobserver visual DS agreement between OSEM and BPL was 0.67 for lesions >1cm and 0.4 for lesions ≤1 cm. F-18 Fluorodeoxyglucose PET/CT of diffuse large B-cell lymphoma reconstructed with BPL has higher SUV values, altered DSs and reader agreement when compared to OSEM. This report finds volumetric PET measurements such as metabolic tumor volume to be similar between BPL and OSEM PET reconstructions. Efforts such as adoption of European Association Research Ltd accreditation should be made to harmonize PET data with an aim at balancing the need for harmonization and sensitivity for lesion detection.

## 1. Introduction

Positron emission tomography (PET) image reconstruction has evolved from filtered backprojection to iterative methods such as ordered subset expectation maximization (OSEM), to more recent methods like point spread function (PSF) and Bayesian penalized likelihood (BPL) reconstruction algorithms such as Q.Clear (GE Healthcare, Waukesha, WI).^[1]^ Q.Clear utilizes PSF modeling while taking input from surrounding voxels as a penalty for increasingly higher levels of noise inherit within subsequent image reconstruction iterations. The penalty factor (*β*) is adjustable and allows users to tune the level of image noise.

Phantom spatial resolution of PSF and BPL reconstruction has been reported higher than OSEM with regard to objects smaller than 1 cm.^[2–8]^ Some data point to BPL having higher spatial resolution when compared to PSF.^[8]^ Clinically, BPL reconstruction reduces noise, especially in larger patients and within smaller lesions.^[9–15]^

The maximum standard uptake value (SUVmax) body weight (BW, SUVmax) is a semi-quantitative metric for intensity of radiotracer activity within a region of interest accounting for amount of injected dose and BW while correcting for radioactive time decay [$SUVmax=\frac{ROI}{ID/BW}$]. Typically, SUVmax values of PSF and BPL are higher than OSEM (especially in smaller lesions) and this difference tends to be more pronounced in tumors relative to normal liver.^[14–21]^ Alterations in relative SUVmax values, and the relationship between lesion SUV compared to liver, raise concern around accuracy and consistency from interpreting physicians, including when imaging lymphoma.^[22]^

Reader agreement using OSEM reconstructed PET data are generally moderate to high.^[23–27]^ Prior studies have shown moderate interobserver agreement between the visual Deauville scores (DS) when imaging diffuse large B-cell lymphoma (DLBCL) with OSEM reconstructed F-18 fluorodeoxyglucose (FDG) PET exams.^[28–31]^ However, there is sparce data on intra and interobserver agreement between different methods of PET reconstruction when imaging DLBCL. Here we assess the difference between OSEM and BPL (Q.Clear) regarding PET computed tomography (CT) quantitative metrics and DS reader agreement within a group of patients with DLBCL.

## 2. Methods

### 2.1. Patients

After approval by the Mayo Clinic Institutional Review Board, a single institution retrospective review was initiated by running a query within a single tertiary referral center patient database searching for sequential adult patients with exam indications containing the terms “DLBCL” and “B cell” between January 1, 2016 and August 8, 2018.

### 2.2. PET/CT technique

Patients were instructed to follow a dietary fast for at least 4 hours prior to the exam. Exams were performed using standard PET/CT equipment (GE Discovery 710 and GE Discovery MI) with a clinical oncology technique (PET: 3–5-minute acquisition per bed based on patient body mass index, 192 × 192 matrix, 70 cm field of view. CT: non-enhanced, modulated ~90 mAs, 120 kVp, pitch 1.0, 3.75–5.0 mm slice thickness). The PET exams were reconstructed using both OSEM and BPL (Q.Clear) techniques. BPL reconstruction utilizes time-of-flight and PSF information. The *β* value used for BPL reconstruction was 300.

### 2.3. PET/CT interpretation

Five board-certified Nuclear Radiologists with 3 (JRY), 3 (ECE), 3 (ATP), 9 (ACH), and 24 (MAN) years of clinical experience reviewed the cases using the same standard clinical interpretation hardware and software. One Radiologist moderator (JRY) reviewed the cases and saved sessions with annotations highlighting the most FDG avid lesion >1 cm in addition to the most FDG avid lesion 1 cm and less when present for both the OSEM and BPL reconstructions. The remaining 4 Radiologists served as blinded reviewers. The moderator used MIM Software Inc. (Cleveland, OH), to segment the lesions using an automated method (PET Edge) with resulting BW SUVmax, SUVmean, metabolic tumor volume (MTV) and total lesion glycolysis (TLG) measured for both OSEM and BPL PET reconstructions. Lesions were measured manually for size using the CT images from the PET/CT exam. Physiologic uptake in the liver and thoracic aorta blood pool were measured using 3 cm and 1.5 cm sphere regions of interest, respectively. Signal to noise ratio of the reconstructions were calculated as mean SUV divided by the standard deviation of the SUV in the liver region.

The OSEM and BPL sessions were randomized and distributed to the reviewers in increments of 10 sessions spaced out by at least 2 weeks. Prior to interpretation, all Radiologists underwent a training session where it was agreed to provide DSs visually using the rotating maximum intensity projection images. DSs were to be provided using the Lugano classification where a score of 1 (DS1) is no abnormal uptake, a score of 2 (DS2) is uptake less than or equal to mediastinal blood pool, a score of 3 (DS3) is uptake between mediastinal blood pool and liver, a score of 4 (DS4) is uptake moderately greater than liver and a score of 5 (DS5) is uptake markedly higher than liver.^[31,32]^ The Radiologists independently reviewed each session in a blinded fashion.

### 2.4. Statistical analysis

Analysis of the data was performed using SAS (version 9.4, Boston, MA) and BlueSky (version 7.40, Cary, NC). Continuous data is reported as a mean and range or standard deviation. Continuous variables were correlated using a paired *t* test. Categorical data is reported with absolute values and relative frequencies. Interobserver agreement was calculated using Kendall coefficient of concordance and intraobserver agreement was calculated using weighted *κ*. The overall DS among 4 interpreters is reported as the median. A *P* value <.05 is considered statistically significant.

## 3. Results

The initial search produced 223 unique PET/CT exams, of which 112 were reconstructed with both OSEM and BPL (Q.Clear) methods. Five exams were excluded due to the following: irretrievable image data (2), exam from outside institution (2), large amount of radiotracer extravasation (1). Three exams were used for training purposes. A total of 104 unique exams among 52 patients were available for final analysis. Each exam was reconstructed using both OSEM and BPL (Q.Clear) methods producing 208 total sessions for interpretation.

Automated segmentation using PET Edge (MIM Software Inc., Cleveland, OH) required manual input by the moderator for accurate segmentation in 23% (16/69) of lesions >1 cm and 16% (10/62) of lesions ≤1 cm. Inaccurate automatic segmentation requiring manual input was often due to lesions near structures with high physiologic FDG uptake such as the brain, kidneys, and urinary bladder.

Patient demographics, phase of care and physiologic PET related factors are shown in Table 1. Normal liver SUV mean and max values were slightly lower for the BPL compared to OSEM (*P* ≤ .001) while the signal to noise ratio was slightly higher for BPL. Blood pool SUVmean was slightly lower for BPL compared to OSEM (*P* ≤ .001) while there was no significant difference for SUVmax.

**Table 1:** Patient demographics and general PET characteristics.

Patient and PET characteristics	N = 52 patients, 104 exams
Age	
Mean	64.4 yr
Range	19–86 yr
Sex	
Female	21
Male	31
Mean weight (range)	79.7 kg (42–143)
Mean BMI (range)	26.5 kg/m^2^ (17–46)
Phase of care	
Pretreatment	16% (17/104)
Interim	21% (22/104)
End-treatment	18% (19/104)
Other	44% (46/104)
Mean blood glucose (range)	94.9 mg/dL (61–166)
Mean IV FDG activity (range)	14.0 mCi (11.7–16.4)
Mean uptake time (range)	63.3 min (59.3–87.3)
FDG avid lesion >1cm	66% (69/104)
FDG avid lesion ≤1cm	60% (62/104)
No FDG avid lesions	18% (19/104)
Mean lesion size	
>1 cm	4.1 cm (1.2–18.6)
≤1 cm	0.8 cm (0.3–1.0)
Liver mean SUVmean	
OSEM	2.39 ± 0.38 g/mL, SNR = 10.13*
BPL	2.29 ± 0.38 g/mL, SNR = 10.28*
Difference	−3.8% (95% CI: −0.11, −0.07), ***P* < .001**
Liver mean SUVmax	
OSEM	3.14 (SD = 0.54)
BPL	2.98 (SD = 0.48)
Difference	−5.1% (95% CI: −0.22, −0.01), ***P* < .001**
Blood pool mean SUVmean	
OSEM	1.83 (SD = 0.28)
BPL	1.78 (SD = 0.32)
Difference	−3.3% (95% CI: −0.09, −0.02), ***P* < .001**
Blood pool mean SUVmax	
OSEM	2.20 (SD = 0.37)
BPL	2.16 (SD = 0.39)
Difference	−1.8% (95% CI: −0.09, 0.02), *P* = .16

BMI = body mass index, BPL = Bayesian penalized likelihood, CI = confidence interval, FDG = F-18 fluorodeoxyglucose, OSEM = ordered subset expectation maximization, PET = positron emission tomography, SD = standard deviation, SNR = signal to noise ratio, SUV = standard uptake value.

* Mean signal-to-noise ratio calculated per patient as liver SUVmean divided by the SUVmean standard deviation.

The differences between OSEM and BPL quantitative PET metrics for FDG avid lesions are depicted in Tables 2 and 3 with stratification between lesion diameters >1 cm and those ≤1 cm. For both lesion sizes, SUV max and mean values were significantly (*P* < .001) greater for BPL versus OSEM reconstructions. Of all lesions ≥1 cm, BPL had a mean 26% greater SUVmax compared to OSEM. Of all lesions ≤1 cm, BPL had a mean 112% greater SUVmax compared to OSEM. For lesions >1 cm, BPL shifted 13% of SUVmax values from less than, to greater than liver. For lesions ≤1 cm, BPL shifted 34% of SUVmax values from less than, to greater than liver. There was no significant difference between BPL and OSEM for TLG or MTV in lesions >1 cm. Lesions ≤1 cm had significantly lower MTV by 52% when reconstructed with BPL compared to OSEM (*P* < .001). The lesion-to-liver SUVmax ratios were 36% and 132% higher with BPL compared to OSEM for lesions >1 cm and ≤1 cm, respectively.

**Table 2:** Quantitative PET metrics for lymphomatous lesions >1 cm in diameter.

Lesion >1 cm	n = 69
Mean SUVmax	
OSEM	11.7 (SD = 10.5)
BPL	14.7 (SD = 12.4)
Difference	26% (95% CI: 2.39, 3.64), ***P* < .001**
Mean SUVmean	
OSEM	6.5 (SD = 5.8)
BPL	7.2 (SD = 6.0)
Difference	11% (95% CI: 0.51, 0.96), ***P* < .001**
Mean MTV	
OSEM	94.2 (SD = 250.9)
BPL	94.8 (SD = 267.1)
Difference	≤1% (95% CI: −9.44, 10.53), *P* = .913
Mean TLG	
OSEM	735.7 (SD = 1753.5)
BPL	742.1 (SD = 1865.3)
Difference	≤1% (95% CI: −66.03, 78.95), *P* = .860
Mean lesion to liver SUVmax	
OSEM	4.03 (SD = 3.61)
BPL	5.47 (SD = 4.76)
Difference	36% (95% CI: 0.70–1.21), ***P* < .001**

BPL = Bayesian penalized likelihood, CI = confidence interval, FDG = F-18 fluorodeoxyglucose, MTV = metabolic tumor volume, OSEM = ordered subset expectation maximization, PET = positron emission tomography, SD = standard deviation, SUV = standard uptake value, TLG = total lesion glycolysis.

**Table 3:** Quantitative PET metrics for lymphomatous lesions ≤1 cm in diameter.

Lesion ≤1 cm	n = 62
Mean SUVmax	
OSEM	5.0 (SD = 5.0)
BPL	10.7 (SD = 9.8)
Difference	112% (95% CI: 4.00, 7.22), ***P* < .001**
Mean SUVmean	
OSEM	3.8 (SD = 3.8)
BPL	7.6 (SD = 6.8)
Difference	100% (95% CI: 2.71, 4.93), ***P* < .001**
Mean MTV	
OSEM	1.3 (SD = 1.3)
BPL	0.6 (SD = 1.2)
Difference	−52% (95% CI: −1.03, −0.32), ***P* < .001**
Mean TLG	
OSEM	4.2 (SD = 4.2)
BPL	3.7 (SD = 6.1)
Difference	−13% (95% CI: −1.23, 0.16), *P* = .131
Mean lesion to liver SUVmax	
OSEM	1.70 (SD = 1.43)
BPL	3.95 (SD = 3.76)
Difference	132% (95% CI: 0.88–1.82), ***P* < .001**

BPL = Bayesian penalized likelihood, CI = confidence interval, MTV = metabolic tumor volume, OSEM = ordered subset expectation maximization, PET = positron emission tomography, SD = standard deviation, SUV = standard uptake value, TLG = total lesion glycolysis.

Changes in visual DS and reader agreement between OSEM and BPL are shown in Table 4. To explore possible clinical implications, the highest DS between lesions >1 cm and ≤1 cm was utilized for each exam and the median DS between the interpreting Radiologists was used as a final, summed DS. There was no DS change in 75% (78/104) of cases. However, 23% (24/104) of cases were upgraded with higher DS with BPL compared to OSEM. Of the upgraded cases, 9% (9/104) were shifted into the DS4/DS5 range. While 1.9% (2/104) were downgraded into the DS1 and DS2 range. The interobserver agreement among the 4 readers was very good to excellent for both OSEM (0.81–0.89) and BPL (0.75–0.85) PET reconstructions. The intraobserver agreement between OSEM and BPL was good to very good (0.63–0.73) for lesions >1 cm and fair (0.29–0.44) for lesions ≤1 cm.

**Table 4:** PET visual Deauville score (DS) observer agreement.

FDG PET/CT DS interpretation	Observer agreement (95% CI)
Lesions >1 cm DS interobserver agreement*	
OSEM	0.89 (0.85, 0.92)
BPL	0.85 (0.78, 0.90)
Lesions ≤1 cm DS interobserver agreement*	
OSEM	0.84 (0.77, 0.89)
BPL	0.81 (0.74, 0.87)
OSEM & BPL DS intraobserver agreement†	
Reader 1	
Lesion >1 cm	0.64 (0.50, 0.79)
Lesion ≤1 cm	0.38 (0.24, 0.52)
Reader 2	
Lesion >1 cm	0.68 (0.56, 0.80)
Lesion ≤1 cm	0.29 (0.14, 0.44)
Reader 3	
Lesion >1 cm	0.73 (0.63, 0.83)
Lesion ≤1 cm	0.42 (0.29, 0.55)
Reader 4	
Lesion >1 cm	0.63 (0.51, 0.75)
Lesion ≤1cm	0.44 (0.30, 0.59)
All 4 readers	
Lesion >1 cm	0.67 (0.61, 0.74)
Lesion ≤1cm	0.40 (0.33, 0.47)
Overall visual DS	(Absolute values)
OSEM	DS1:44% (46/104), DS2:5% (5/104), DS3:10% (10/104), DS4:13% (13/104), DS5:29% (30/104)
BPL	DS1:38% (40/104), DS2:3% (3/104), DS3:9% (9/104), DS4:13% (14/104), DS5:37% (38/104)
Overall visual DS OSEM to BPL shift‡	(absolute values)
Downgrade: OSEM DS3 to BPL DS2	0.9% (1/104)
Downgrade: OSEM DS2 to BPL DS1	0.9% (1/104)
No change: OSEM DS = BPL DS	75% (78/104)
Upgrade: OSEM DS1 to BPL DS3	4.8% (5/104)
Upgrade: OSEM DS1 to BPL DS4	1.9% (2/104)
Upgrade: OSEM DS2 to BPL DS3	1.9% (2/104)
Upgrade: OSEM DS3 to BPL DS4	6.7% (7/104)
Upgrade: OSEM DS4 to BPL DS5	7.7% (8/104)
Overall OSEM to BPL difference	0.30 (95% CI: 0.17–0.43), *P* < .001

BPL = Bayesian penalized likelihood, CI = confidence interval, CT = computed tomography, DS = Deauville scores, FDG = F-18 fluorodeoxyglucose, OSEM = ordered subset expectation maximization, PET = positron emission tomography.

* Interobserver agreement calculated with Kendall coefficient of concordance.

† Intraobserver agreement calculated with weighted kappa.

‡ Overall DS is the highest score of the lesions > and ≤1 cm.

## 4. Discussion

Debate surrounds differences between OSEM and BPL PET reconstruction methods when determining DS.^[22,33]^ This study found significantly higher SUVmax and SUVmean values when measured with BPL compared to OSEM. The difference between BPL and OSEM for volumetric PET data (MTV and TLG) was only significant for MTV when lesions were <1 cm. The visual DS within individual patients was changed in 25% of cases when OSEM and BPL was reviewed by the same reader. Interobserver visual DS agreement was good to excellent for both OSEM and BPL. However, intraobserver visual DS agreement between OSEM and BPL PET reconstructions was fair.

**Figure 1.**
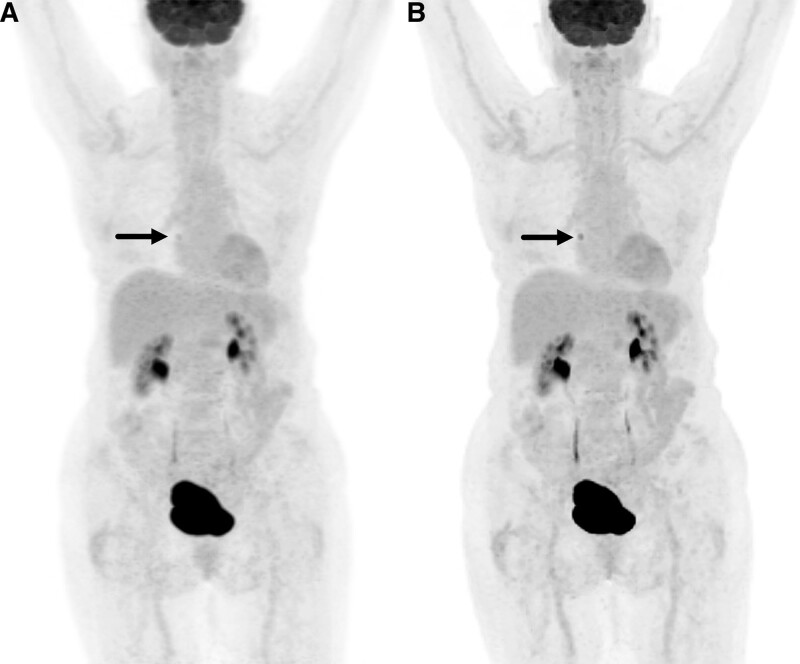
Eighty-year-old female with diffuse large B-cell lymphoma status post 6 cycles of R-CHOP chemotherapy. Maximum intensity projection F-18 fluorodeoxyglucose positron emission tomography highlighting a mediastinal lesion (arrows) that shifted from a Deauville score of 3 to 4 when reconstructed with (A) OSEM versus (B) BPL respectively. BPL = Bayesian penalized likelihood, OSEM = ordered subset expectation maximization, R-CHOP = .

Our findings within a population of DLBCL patients align with prior reports of higher BPL SUV values compared to OSEM PET reconstruction in a variety of tumors.^[14–21]^ Lymphoma lesions are often evaluated relative to normal liver and blood pool FDG uptake.^[31,32]^ A rise in lesion SUV values with BPL could be controlled by reporting lesion-to-liver SUV ratios and methods as such have been proposed.^[34]^ However, lesion-to-liver SUVmax ratios within our study had an average BPL ratio of 5.5 and OSEM ratio of 4.0 for lesions >1cm, with more drastic differences for lesions ≤1 cm with an average BPL ratio of 4.0 and OSEM ratio of 1.7. Therefore, caution should be used when using BPL SUVmax values for staging DLBCL, especially in lesions ≤1 cm. That said, volumetric PET data seems somewhat immune to differences between BPL and OSEM reconstruction methods. There were no significant differences between MTV and TLG for lesions >1 cm and only a significant difference in MTV for lesions ≤1 cm. However, differences in tumor volumes <1 cc are of questionable clinical significance such as what is shown in Figure 1. These findings suggest adoption of volumetric PET data may improve consistency of results when imaging DLBCL between different PET reconstruction techniques.

In 2018 Enilorac et al found a minor risk of altering DS or change in clinical outcomes of lymphoma patients when comparing PSF with a European Association Research Ltd (EARL) harmonized filter.^[35]^ Enilorac et al used quantitative SUV thresholds for obtaining DS and when DS was grouped into DS1 to DS3 and DS4 to DS5 categories, the frequency of discordance was 3.2% at end of treatment and 5.0% at interim exams. In 2020 Wyrzykowski et al evaluated the impact of BPL (Q.Clear) compared to OSEM on lymphoma DS using lesion SUVmax to normal liver/ blood pool thresholding and found an overall discordance of 15.7% with 7.1% converting to DS4/DS5 with BPL.^[36]^ The visual DS method of this study found an overall discordance of 25% when using the median DS of the 4 Radiologists. Our group had statistically significant elevations of DS with BPL compared to OSEM, most shifted from DS4 to DS5 (7.7%) which may not be clinically significant. However, 6.7% (7/104) shifted from DS3 to DS4 and 1.9% (2/104) shifted from DS1 to DS4 with BPL compared to OSEM which is more likely to impact patient care.

Previously reported DS interobserver FDG PET/CT agreement has ranged from 0.35 to 0.87 with evidence for improvements when using a training session prior to data collection.^[28–30]^ We report a slightly higher agreement for OSEM compared to BPL with Kendall coefficient of concordance scores of 0.81 and 0.75, respectively. Our interobserver agreement is at the high end of prior reports which may be attributable to the training session incorporated into our method.

Intraobserver FDG PET/CT visual DS agreement when interpreted with and without clinical information has been reported at 0.48 and 0.62, respectively.^[30]^ Among our 4 reviewers, the average intraobserver agreement between OSEM and BPL was 0.67 (0.63–0.73) for lesions >1 cm and 0.40 (0.29–0.44) for lesions ≤1 cm (weighted *κ*). While our results are similar to prior reports for lesions >1 cm, the intraobserver agreement for lesions ≤1 cm is lower. Therefore, greater variation between OSEM and BPL reconstruction results may be expected when interpreting small lymphomatous lesions.

There are weaknesses inherent to this retrospective review. A small to moderate number of patients (52) and exams (104) resulted for review. There were 18% of exams without FDG avid lesions which may increase observer agreement. However, this rate mimics clinical practice where not all exams are positive. The intra-observer agreement between OSEM and BPL exams was subject to recency bias. However, reviewers were blinded, interpreted exams randomly and in increments of 10 sessions spaced out by 2-week intervals to help minimize fatigue and recency bias. That said, there may be less clinical relevance of this review due to the blinded method and inclusion of lesions ≤1 cm which may be deemed inconsequential clinically. However, there is evidence pointing to little impact of clinical information on observer agreement in this context.^[30]^ Further, to ensure observers were interpreting the exact same lesion, a session was saved within the reviewing software annotating lesions for DS assignment which is subject to leading bias.

Currently, a mix of PET technologies in practice pose a challenge to clinical care and research. A common solution is producing two PET reconstructions: harmonized for quantitative measurement and optimized for lesion detection. Various organizations have worked towards PET standardization among different vendors including the European Association of Nuclear Medicine which launched the EARL accreditation program in 2006. The first generation of PET accreditation (EARL1) was initiated in 2010 and updated in 2015.^[37,38]^ Recognizing the need to account for emerging advanced PET techniques such as time-of-flight and PSF reconstruction, EARL2 was developed and validated.^[39]^ Becoming accredited with EARL will minimize differences in quantitative PET data. Additionally, PET reader agreement can be improved with PET harmonization.^[29,40]^ Progress in clinical PET harmonization has been made.^[41]^ However, there is a persistent challenge of transitioning to more advanced PET techniques.

## 5. Conclusion

Growing evidence point to a relevant DS shift between OSEM and BPL PET reconstruction methods when imaging lymphoma with FDG, yet volumetric PET data such as MTV and TLG seem less prone to different PET techniques. While interobserver agreement between DS is high for both OSEM and BPL, the intraobserver agreement suffers for smaller lesions. PET harmonization using EARL accreditation methods is encouraged to optimize patient care and research. Acknowledgments

The authors acknowledge statistical methods consultation from the Biostatistics, Epidemiology and Research Design resource of Mayo Clinic’s Center for Clinical and Translational Science, and would like to thank Sonia Watson, PhD, for assistance in preparation of the manuscript.

## Author contributions

**Conceptualization:** Jason R Young, Vamshi K Mugu, Geoffrey B Johnson, Eric C Ehman, Annie T Packard, Andrew C Homb, Mark A Nathan, Gita Thanarajasingam, Bradley J Kemp.

**Data curation:** Jason R Young, Vamshi K Mugu, Eric C Ehman, Annie T Packard, Andrew C Homb, Mark A Nathan, Bradley J Kemp.

**Formal analysis:** Jason R Young, Vamshi K Mugu, Eric C Ehman, Annie T Packard, Andrew C Homb, Mark A Nathan, Gita Thanarajasingam, Bradley J Kemp.

**Investigation:** Jason R Young, Vamshi K Mugu, Geoffrey B Johnson, Eric C Ehman, Annie T Packard, Andrew C Homb, Mark A Nathan, Gita Thanarajasingam, Bradley J Kemp.

**Methodology:** Jason R Young, Vamshi K Mugu, Geoffrey B Johnson, Eric C Ehman, Annie T Packard, Andrew C Homb, Mark A Nathan, Gita Thanarajasingam, Bradley J Kemp.

**Project administration:** Geoffrey B Johnson, Bradley J Kemp.

**Supervision:** Jason R Young, Geoffrey B Johnson, Annie T Packard, Andrew C Homb, Mark A Nathan, Bradley J Kemp.

**Validation:** Jason R Young, Vamshi K Mugu, Andrew C Homb, Bradley J Kemp.

**Writing – original draft:** Jason R Young, Bradley J Kemp.

**Writing – review & editing:** Jason R Young, Vamshi K Mugu, Geoffrey B Johnson, Eric C Ehman, Annie T Packard, Andrew C Homb, Mark A Nathan, Gita Thanarajasingam, Bradley J Kemp.

## References

[1] Ross S. Q.Clear White Paper. General Electric Healthcare website. 2014. Available at: https://wwwgehealthcarecouk/-/jssmedia/widen/gehealthcarecom/migrated/ocuments-us-global-products-pet-ct-whitepaper-q-clear-ge-healthcare-white-paper_qclear_pdfpdf?rev=2ffc6f8f38a542ab90c75d37f4ac7183&hash=076FF90CE4A7F9D77E2C544FFAE695DA. Updated July 25, 2019 [Access date November 5, 2021].

[2] Teoh EJ McGowan DR Macpherson RE. Phantom and clinical evaluation of the Bayesian penalized likelihood reconstruction algorithm Q.Clear on an LYSO PET/CT System. J Nucl Med. 2015;56:1447–52. 26159585 10.2967/jnumed.115.159301 PMC4558942

[3] Bettinardi V Presotto L Rapisarda E. Physical performance of the new hybrid PETCT Discovery-690. Med Phys. 2011;38:5394–411. 21992359 10.1118/1.3635220

[4] Alessio AM Stearns CW Tong S. Application and evaluation of a measured spatially variant system model for PET image reconstruction. IEEE Trans Med Imaging. 2010;29:938–49. 20199927 10.1109/TMI.2010.2040188 PMC2903538

[5] van der Vos CS Koopman D Rijnsdorp S. Quantification, improvement, and harmonization of small lesion detection with state-of-the-art PET. Eur J Nucl Med Mol Imaging. 2017;44:4–16. 28687866 10.1007/s00259-017-3727-z PMC5541089

[6] Miwa K Wagatsuma K Nemoto R. Detection of sub-centimeter lesions using digital TOF-PET/CT system combined with Bayesian penalized likelihood reconstruction algorithm. Ann Nucl Med. 2020;34:762–71. 32623569 10.1007/s12149-020-01500-8

[7] Texte E Gouel P Thureau S. Impact of the Bayesian penalized likelihood algorithm (Q.Clear(R)) in comparison with the OSEM reconstruction on low contrast PET hypoxic images. EJNMMI Phys. 2020;7:28. 32399752 10.1186/s40658-020-00300-3 PMC7218037

[8] Rogasch JM Suleiman S Hofheinz F. Reconstructed spatial resolution and contrast recovery with Bayesian penalized likelihood reconstruction (Q.Clear) for FDG-PET compared to time-of-flight (TOF) with point spread function (PSF). EJNMMI Phys. 2020;7:2. 31925574 10.1186/s40658-020-0270-y PMC6954158

[9] Chilcott AK Bradley KM McGowan DR. Effect of a Bayesian penalized likelihood PET reconstruction compared with ordered subset expectation maximization on clinical image quality over a wide range of patient weights. AJR Am J Roentgenol. 2018;210:153–7. 29091008 10.2214/AJR.17.18060

[10] Lindstrom E Sundin A Trampal C. Evaluation of penalized-likelihood estimation reconstruction on a digital time-of-flight PET/CT scanner for (18)F-FDG whole-body examinations. J Nucl Med. 2018;59:1152–8. 29449445 10.2967/jnumed.117.200790

[11] Vallot D Caselles O Chaltiel L. A clinical evaluation of the impact of the Bayesian penalized likelihood reconstruction algorithm on PET FDG metrics. Nucl Med Commun. 2017;38:979–84. 29045338 10.1097/MNM.0000000000000729

[12] Sampaio Vieira T Borges Faria D Azevedo Silva F. The impact of a Bayesian penalized likelihood reconstruction algorithm on the evaluation of indeterminate pulmonary nodules by dual-time point 18F-FDG PET/CT. Clin Nucl Med. 2017;42:e352–4. 28525459 10.1097/RLU.0000000000001713

[13] Sampaio Vieira T Borges Faria D Azevedo Silva F. The impact of a Bayesian penalized-likelihood reconstruction algorithm on delayed-time-point Ga-68-PSMA PET for improved recurrent prostate cancer detection. Eur J Nucl Med Mol Imaging. 2018;45:1461–2. 29679111 10.1007/s00259-018-4023-2 PMC5993856

[14] Kurita Y Ichikawa Y Nakanishi T. The value of Bayesian penalized likelihood reconstruction for improving lesion conspicuity of malignant lung tumors on (18)F-FDG PET/CT: comparison with ordered subset expectation maximization reconstruction incorporating time-of-flight model and point spread function correction. Ann Nucl Med. 2020;34:272–9. 32060780 10.1007/s12149-020-01446-x

[15] Howard BA Morgan R Thorpe MP. Comparison of Bayesian penalized likelihood reconstruction versus OS-EM for characterization of small pulmonary nodules in oncologic PET/CT. Ann Nucl Med. 2017;31:623–8. 28689358 10.1007/s12149-017-1192-1

[16] Quak E Hovhannisyan N Lasnon C. The importance of harmonizing interim positron emission tomography in non-Hodgkin lymphoma: focus on the Deauville criteria. Haematologica. 2014;99:e84–5. 24584350 10.3324/haematol.2014.104125 PMC4040900

[17] Kuhnert G Boellaard R Sterzer S. Impact of PET/CT image reconstruction methods and liver uptake normalization strategies on quantitative image analysis. Eur J Nucl Med Mol Imaging. 2016;43:249–58. 26280981 10.1007/s00259-015-3165-8

[18] Matti A Lima GM Pettinato C. How do the more recent reconstruction algorithms affect the interpretation criteria of PET/CT images? Nucl Med Mol Imaging. 2019;53:216–22. 31231442 10.1007/s13139-019-00594-x PMC6554360

[19] Messerli M Stolzmann P Egger-Sigg M. Impact of a Bayesian penalized likelihood reconstruction algorithm on image quality in novel digital PET/CT: clinical implications for the assessment of lung tumors. EJNMMI Phys. 2018;5:27. 30255439 10.1186/s40658-018-0223-x PMC6156690

[20] Teoh EJ McGowan DR Bradley KM. Novel penalised likelihood reconstruction of PET in the assessment of histologically verified small pulmonary nodules. Eur Radiol. 2016;26:576–84. 25991490 10.1007/s00330-015-3832-y PMC4551414

[21] Parvizi N Franklin JM McGowan DR. Does a novel penalized likelihood reconstruction of 18F-FDG PET-CT improve signal-to-background in colorectal liver metastases? Eur J Radiol. 2015;84:1873–8. 26163992 10.1016/j.ejrad.2015.06.025

[22] Barrington SF Sulkin T Forbes A. All that glitters is not gold - new reconstruction methods using Deauville criteria for patient reporting. Eur J Nucl Med Mol Imaging. 2018;45:316–7. 29198033 10.1007/s00259-017-3893-z

[23] Teoh EJ McGowan DR Schuster DM. Bayesian penalised likelihood reconstruction (Q.Clear) of (18)F-fluciclovine PET for imaging of recurrent prostate cancer: semi-quantitative and clinical evaluation. Br J Radiol. 2018;91:20170727. 29303359 10.1259/bjr.20170727 PMC6190769

[24] Toriihara A Nobashi T Baratto L. Comparison of 3 interpretation criteria for (68)Ga-PSMA11 PET based on inter- and intrareader agreement. J Nucl Med. 2020;61:533–9. 31562226 10.2967/jnumed.119.232504

[25] Gultekin A Yaylali O Sengoz T. Intraobserver and interobserver agreement for the interpretation of 68Ga-prostate-specific membrane antigen-I&T positron emission tomography/computed tomography imaging. Nucl Med Commun. 2019;40:1250–5. 31584465 10.1097/MNM.0000000000001097

[26] Zacho HD Fonager RF Nielsen JB. Observer agreement and accuracy of (18)F-sodium fluoride PET/CT in the diagnosis of bone metastases in prostate cancer. J Nucl Med. 2020;61:344–9. 31481577 10.2967/jnumed.119.232686 PMC7067525

[27] Xiao J Wang D Guo B. Observer agreement and accuracy of 18F-sodium fluoride PET/computed tomography in the diagnosis of skull-base bone invasion and osseous metastases in newly diagnosed nasopharyngeal carcinoma. Nucl Med Commun. 2020;41:942–9. 32796483 10.1097/MNM.0000000000001243

[28] Han EJ O JH Yoon H. FDG PET/CT response in diffuse large B-cell lymphoma: Reader variability and association with clinical outcome. Medicine (Baltim). 2016;95:e4983. 10.1097/MD.0000000000004983 PMC5265944 27684851

[29] Ceriani L Barrington S Biggi A. Training improves the interobserver agreement of the expert positron emission tomography review panel in primary mediastinal B-cell lymphoma: interim analysis in the ongoing International Extranodal Lymphoma Study Group-37 study. Hematol Oncol. 2017;35:548–53. 27545416 10.1002/hon.2339

[30] Arimoto MK Nakamoto Y Higashi T. Intra- and inter-observer agreement in the visual interpretation of interim 18F-FDG PET/CT in malignant lymphoma: influence of clinical information. Acta Radiol. 2018;59:1218–24. 29333861 10.1177/0284185117751279

[31] Cheson BD Fisher RI Barrington SF. Recommendations for initial evaluation, staging, and response assessment of Hodgkin and non-Hodgkin lymphoma: the Lugano classification. J Clin Oncol. 2014;32:3059–67. 25113753 10.1200/JCO.2013.54.8800 PMC4979083

[32] Barrington SF Mikhaeel NG Kostakoglu L. Role of imaging in the staging and response assessment of lymphoma: consensus of the International Conference on Malignant Lymphomas Imaging Working Group. J Clin Oncol. 2014;32:3048–58. 25113771 10.1200/JCO.2013.53.5229 PMC5015423

[33] Bradley KM McGowan DR Gleeson FV. Embrace progress. J Nucl Med. 2018;59:1169–1169. 29700130 10.2967/jnumed.118.212761

[34] Barrington SF Kluge R. FDG PET for therapy monitoring in Hodgkin and non-Hodgkin lymphomas. Eur J Nucl Med Mol Imaging. 2017;44:97–110. 28411336 10.1007/s00259-017-3690-8 PMC5541086

[35] Enilorac B Lasnon C Nganoa C. Does PET reconstruction method affect Deauville score in lymphoma patients? J Nucl Med. 2018;59:1049–55. 29242403 10.2967/jnumed.117.202721

[36] Wyrzykowski M Siminiak N Kazmierczak M. Impact of the Q.Clear reconstruction algorithm on the interpretation of PET/CT images in patients with lymphoma. EJNMMI Res. 2020;10:99. 32845406 10.1186/s13550-020-00690-6 PMC7450027

[37] Boellaard R O’Doherty MJ Weber WA. FDG PET and PET/CT: EANM procedure guidelines for tumour PET imaging: version 1.0. Eur J Nucl Med Mol Imaging. 2010;37:181–200. 19915839 10.1007/s00259-009-1297-4 PMC2791475

[38] Boellaard R Delgado-Bolton R Oyen WJ. FDG PET/CT: EANM procedure guidelines for tumour imaging: version 2.0. Eur J Nucl Med Mol Imaging. 2015;42:328–54. 25452219 10.1007/s00259-014-2961-x PMC4315529

[39] Kaalep A Burggraaff CN Pieplenbosch S. Quantitative implications of the updated EARL 2019 PET-CT performance standards. EJNMMI Phys. 2019;6:28. 31879795 10.1186/s40658-019-0257-8 PMC6933045

[40] Nestle U Rischke HC Eschmann SM. Improved inter-observer agreement of an expert review panel in an oncology treatment trial--Insights from a structured interventional process. Eur J Cancer. 2015;51:2525–33. 26277100 10.1016/j.ejca.2015.07.036

[41] Aide N Lasnon C Veit-Haibach P. EANM/EARL harmonization strategies in PET quantification: from daily practice to multicentre oncological studies. Eur J Nucl Med Mol Imaging. 2017;44:17–31. 10.1007/s00259-017-3740-2 PMC5541084 28623376

